# Editorial: Natural products in regulating mitochondrial dysfunction

**DOI:** 10.3389/fphar.2023.1233718

**Published:** 2023-06-30

**Authors:** Caifeng Xie, Liang Ma, Xin Wang, Xiangyang Xiong

**Affiliations:** ^1^ School of Basic Medical Sciences, Nanchang University, Nanchang, China; ^2^ Division of Nephrology, Kidney Research Institute, West China Hospital of Sichuan University, Chengdu, China; ^3^ Key Laboratory of Molecular Biology and Gene Engineering of Jiangxi Province, School of Life Sciences, Nanchang University, Nanchang, China; ^4^ Graduate School of Agriculture, Hokkaido University, Sapporo, Japan

**Keywords:** natural products, mitochondrial dysfunction, metabolism, oxidative stress, mitochondrial disease

Mitochondria plays a central role in energy production for all multicellular eukaryotes by synthesizing ATP and maintaining metabolic homeostasis. Actually, these fantastic organelles perform multifunction for the cell beyond simply being a “fuel” (Nunnari and Suomalainen, 2012). Mitochondria not only governs many metabolic processes including oxidative phosphorylation (OXPHOS), electron transport chain (ETC), Krebs cycle, fatty acid oxidation, and many others, but also modulates cell proliferation, cell death, and even inflammatory response (Monzel et al., 2023). Damages caused by mitochondrial DNA (mtDNA) mutations or quality control disorders tend to accumulate in these organelles and led to mitochondrial dysfunction, which is characterized by a loss of efficiency in the electron transport chain and reductions in the synthesis of high-energy molecules. However, recent advances show that mitochondrial dysfunction is being recognized to be even more complex than originally thought (Vafai and Mootha, 2012; Sorrentino et al., 2018). Mitochondrial disease caused by mitochondrial dysfunction is associated with a wide range of human pathologies, such as cancer, metabolic, and cardiovascular diseases, autoimmune diseases, neurobehavioral and psychiatric diseases, neurodegenerative diseases, and so on (Lin and Beal, 2006; Prakash et al., 2017; Chiu et al., 2020).

There is currently no effective treatment for mitochondrial disease, especially for inherited mitochondrial diseases caused by either mtDNA or nuclear DNA mutations. The major management strategy for mitochondrial disease is supportive therapy including nutritional management, exercise, vitamin or amino acid supplements (El-Hattab et al., 2017; Zhong et al., 2022). It is hypothesized that natural products or compounds derived from traditional herbs with antioxidant or metabolic reprogramming capacity could be potential treatment options (Nicolson, 2014; Cho et al., 2019; Mohammadipour, 2022). Thus, it is necessary to explore more mitochondria protective agents, especially natural products, and fully understand the pathophysiology of the disease caused by mitochondrial dysfunction. The present Research Topic aims to provide a platform for communication of current scientific evidence available about the role of natural products in regulating mitochondrial dysfunction.

The present Research Topic features five articles, including one original and four review articles. These articles are dedicated to exploring the mechanism underlying the dysfunction of mitochondria and trying to find the way to ameliorate this disorder by natural products.

Non-alcoholic fatty liver disease (NAFLD) is the most common type of liver disease which finally would lead to liver failure and hepatocellular carcinoma. The mechanism underlying the development and progression of NAFLD is closely related to the dysfunction of mitochondria. Studies suggested that saturated fatty acids or impaired mitochondrial dynamics produce reactive oxygen species and endoplasmatic reticulum stress, which ultimately leads to inflammation, apoptosis, and liver scarring (Longo et al., 2021; Meex and Blaak, 2021). Therefore, targeting mitochondrial dysfunction is probably a potent therapeutic strategy for NAFLD. An original research article (García-Berumen et al.) regarding a novel treatment of NAFLD by restoring mitochondrial function was included in this Research Topic. Avocado oil extracted from the fruit of the avocado tree (Persea americana Mill.) was tested to evaluate its anti-NAFLD effect *in vivo*. The authors demonstrated that avocado oil alleviates NAFLD by attenuating mitochondrial dysfunction, oxidative stress, and inflammation. In this paper, avocado oil showed a protective effect on mitochondrial respiration, complex III activity, and electron transfer in cytochromes in complex III. The present study indicated that regulating mitochondrial function by natural products would be a potential way to ameliorate related diseases, especially metabolic disorders.

Four review papers (Chen et al.; Liu et al.; Tuo et al.; He et al.) were also included in this Research Topic. Chen et al. analyzed the database and summarized the recent advances in the field of mitochondrial dysfunction treated with natural products, and further discussed the role of natural products in modulating mitochondrial quality control systems and regulating mitochondrial functions. The authors concluded that increased research on mitochondria has facilitated the development of new strategies based on natural products to regulate mitochondrial dysfunction. While, at the same time, there are several challenges including dosage optimization, chemical structure modification, and target specificity lie ahead on the road of natural product development. Liu et al. and He et al. discussed the function of natural products in two typical mitochondrial dysfunction-related diseases (metabolic-associated kidney diseases and Parkinson’s disease), respectively. These two papers indicated that natural products were promising to be developed as drugs for the treatment of both metabolic and non-metabolic disorders by restoring mitochondrial dysfunction. However, the efficacy and dosage of natural products are not stable, and the structure-bioactivity relationship of natural products is complex and uncertain. These shortages would limit the application of natural products in the therapy of metabolic-associated kidney diseases and PD. Tuo et al. presented a scoping review on the role of natural products regulating mitochondrial function in cognitive dysfunction. Fourteen studies published from 15 October 2017 to 15 October 2022 were collected, integrated, analyzed, and summarized to assess the therapeutic effect of natural products on cognitive dysfunction. The authors indicated that natural products can improve or reduce cognitive dysfunction by ameliorating mitochondrial dysfunction. But, the limitation of this paper is that few studies were taken into analysis and the results are not so confirmed. Future work should be done to improve the quantity of samples and the quality of the analysis.

Overall, the studies collected in this Research Topic contribute to emphasizing the function of natural products in the regulation of mitochondrial dysfunction. We hope this series of publications will shed light on the intricate mechanisms involved and the development of novel therapeutic strategies for mitochondrial dysfunction.
